# Sustainable Production of Hydrogen by Steam Reforming of Ethanol Using Cobalt Supported on Nanoporous Zeolitic Material

**DOI:** 10.3390/nano10101934

**Published:** 2020-09-28

**Authors:** Javier Francisco da Costa-Serra, Maria Teresa Navarro, Fernando Rey, Antonio Chica

**Affiliations:** Instituto de Tecnología Química, Universitat Politècnica de València-Consejo Superior de Investigaciones Científicas, Avd. de los Naranjos s/n, 46022 Valencia, Spain; jdacosta@itq.upv.es (J.F.d.C.-S.); tnavarro@itq.upv.es (M.T.N.); frey@itq.upv.es (F.R.)

**Keywords:** steam reforming of ethanol, hydrogen, Y zeolite, cobalt catalyst

## Abstract

Cobalt catalysts supported on Y zeolite and mesoporized Y zeolite (Y-mod) have been studied in steam reforming of ethanol (SRE). Specifically, the effect of the mesoporosity and the acidity of the y zeolite as a support has been explored. Mesoporous were generated on Y zeolite by treatment with NH4F and the acidity was neutralized by Na incorporation. Four cobalt catalysts supported on Y zeolite have been prepared, two using Y zeolite without mesoporous (Co/Y, Co/Y-Na), and two using Y zeolite with mesoporous (Co/Y-mod and Co/Y-mod-Na). All catalysts showed a high activity, with ethanol conversion values close to 100%. The main differences were found in the distribution of the reaction products. Co/Y and Co/Y-mod catalysts showed high selectivity to ethylene and low hydrogen production, which was explained by their high acidity. On the contrary, neutralization of the acid sites could explain the higher hydrogen selectivity and the lower ethylene yields exhibited by the Co/Y-Na and Co/Y-mod-Na. In addition, the physicochemical characterization of these catalysts by XRD, BET surface area, temperature-programmed reduction (TPR), and TEM allowed to connect the presence of mesoporous with the formation of metallic cobalt particles with small size, high dispersion, and with high interaction with the zeolitic support, explaining the high reforming activity exhibited by the co/y-mod-Na sample as well as its higher hydrogen selectivity. It has been also observed that the formation of coke is affected by the presence of mesoporous and acidity. Both properties seem to have an opposite effect on the reforming catalyst, decreasing and increasing the coke deposition, respectively.

## 1. Introduction

The continuous increase of the energy consumption, the depletion of fossil fuels, and the environment pollution associated with their use are the three main problems of the current energetic system [1]. According to this panorama, it is clear that it is necessary to find renewable energetic alternatives, cleaner and more sustainable. Hydrogen from renewable sources can be considered as the ultimate clean and climate-neutral energy carrier system. Hydrogen exhibits the greatest heating value (39.4 kWh/kg) of all chemical fuels and its combustion with oxygen produces water, as the only by-product, and no pollutants are generated or emitted. However, hydrogen is not a primary energy source; it must be produced from a primary one. At present the most favorable route to produce hydrogen comes from fossil fuels, mainly through steam reforming of natural gas [2,3], which is associated with the emission of large quantities of greenhouse gases (GHG), especially carbon dioxide (CO2). Consequently, a new eco-friendly hydrogen production route is needed for a clean and sustainable production of energy. Reforming of renewable biomass feedstocks, such as bioethanol, a water and ethanol mixture (10–18 wt. % in ethanol), has become an important and active research area in view of hydrogen production [4,5,6,7]. Bioethanol results in a promising feedstock due to its availability, low toxicity, and easy storage and distribution [8]. It is estimated to have had a worldwide production of 100.2 billion liters in 2016 and it is estimated 134.5 billion liters by 2024 [8]. Thus, catalytic steam reforming of ethanol (SRE) has been intensely investigated for the production of renewable hydrogen [9,10,11,12].

Catalysts play a crucial role in the reactivity toward complete conversion of bioethanol. However, each catalyst induces different reaction pathways and, therefore, the selection of the suitable catalyst is a key factor in the SR of bioethanol. The studies show that the best catalytic results are exhibited by Ni and Co among the non-noble metals [13,14,15,16,17,18,19,20,21,22,23,24,25,26,27,28,29,30,31,32,33,34]. The main problems during the catalytic steam reforming of bioethanol are: (i) sintering of active metal and catalyst poisoning by coke depositing at high temperature, and (ii) formation at moderate temperatures of undesired products such as acetaldehyde, diethyl ether, acetic acid, or ethylene. All these problems were found to be related to the physicochemical properties of the catalyst, which highly depend on: the nature of the metal active site, the preparation methods, the type of metal precursors used, the nature of metal support, the presence of additives, and the operating conditions [32,33,34]. Among them, support plays a crucial role in the preparation of highly active and selective catalysts for the steam reforming of bioethanol, since it favors the dispersion of metal in the catalyst and increases its activity through metal-support interactions [32,33,34]. Specifically, it has been found that the high specific surface of the support may enhance the catalytic activity [16,35], and its topology and the crystal structure may affect the dispersion of the metal particles, improving their stability against sintering [28,36]. Taking this into account, the unique structure of the zeolites would make these materials attractive for use as support of metallic active phases [37,38,39,40]. Considering the above, we believe that the use of Y zeolite, (Faujasite zeolite), as a support of Co, could be an attractive option, since it has a large surface area and can be prepared with neutral characteristics if it is exchanged with alkaline metals. Additionally, mesoporosity can be generated in Y zeolite, which could increase the dispersion and stability of the supported Co metallic particles. Thus, synthesized Y zeolite with neutral characteristics through an ionic exchange with Na could prevent ethanol dehydration reaction. This reaction produces ethylene, a coke precursor, which affects the stability of the catalyst [29,39,40]. The characterization of the cobalt-based catalysts prepared here has been completed and connected to its catalytic performance.

## 2. Materials and Methods

### 2.1. Preparation of Catalysts

Y zeolite was subjected to a dealumination/desilication process for the generation of mesoporous following the methodology reported in [41] with some modifications. Specifically, 5 g of NH_4_-Y zeolite (Si/Al: 2.5, supplied by STREAM) was stirred in 65 mL of aqueous 3M ammonium acetate solution at 298 K for 30 min. Then, 30 g of an aqueous solution of NH_4_F 2.4M was added drop-wise (addition rate: 12 mL/h) on the previous suspension at 353 K under stirring. After complete addition of solution, the suspension was kept stirred for 30 min, followed by filtering and washing with boiling deionized water. The filtered solid was washed in 0.5 L of boiling deionized water for 1 h and then recovered by filtering. This latter procedure was repeated once again. Finally, the result solid was dried at 37 K for 12 h to obtain the final acid zeolite (Y-mod). The neutralization of the acid sites of the samples were accomplished by Na ion exchange. Y and Y-mod zeolites were stirred in a 2.5 M aqueous solution of NaCl (liquid/solid ratio of 10) at 353 K for 1 h. After, the sample was filtered and washed with distilled water and it was dried at 373 K for 30 min. Na ion exchange treatment was repeated 3 times. Finally, the sample was calcined at 773 K for 3 h. Co was incorporated in the Na exchanged and non-exchanged zeolites by incipient wetness impregnation with an aqueous solution containing the required amount of Co(NO_3_)_2_·6H_2_O to achieve a nominal concentration of 20 wt.% of metal in the final catalysts. In this method, the metal precursor was dissolved in a certain volume of milli-Q water, causing the introduction of the metal in the catalyst support due to the capillary action, which causes absorption of the solution into the pores. Finally, the obtained solid was further dried at room temperature for 16 h and, afterwards, calcined in a muffle oven at 873 K for 3 h. Following this procedure, the cobalt-based catalysts were obtained, and they were labelled as Co/Y, Co/Y-Na, Co/Y-mod, and Co/Y-mod-Na.

### 2.2. Characterization Techniques

Inductively coupled plasma with an optical emission spectrometer (Varian 700-ES Series) was used to determine the content of Co, Si, and Al in the support and catalytic materials studied here. Textural properties of the supports and catalysts were obtained from the nitrogen adsorption isotherms determined at 77 K in Micromeritics ASAP 2420 equipment. X-ray diffraction was used to identify the crystalline cobalt oxide and metallic cobalt phases. XRD patterns were obtained at room temperature in a Philips X’pert diffractometer using monochromatized CuKα radiation. The reduction behaviors of the supported oxidized cobalt phases were studied by temperature programmed reduction (TPR) in Micromeritics Autochem 2910 equipment. Fifty mg of the calcined catalyst was initially flushed with 30 cm^3^·min^−1^ of Ar at room temperature for 30 min and then a mixture of 10 vol. % of H_2_ in Ar was passed through the catalyst at a total flow rate of 50 cm^3^·min^−1^ while the temperature was increased up to 1173 K at a heating rate of 10 K·min^−1^. The H_2_ consumption rate was monitored in a thermal conductivity detector (TCD), previously calibrated using the reduction of CuO as reference. Transmission electron microscopy (TEM) micrographs was used to observe the aspect of the fresh and modified Y zeolite supports using a Philips CM-10 microscope operating at 100 kV. Acidity was measured with a Nicolet 710 FTIR spectrometer. Pyridine adsorption–desorption experiments were carried out on self-supported wafers (10 mg·cm^−2^) of calcined samples, previously activated at 673 K and 10^−2^ Pa overnight in a Pyrex vacuum cell fitted with CaF_2_ windows. After activation, the reference spectrum was recorded and pyridine vapor (6.5 × 10^2^ Pa) was admitted in the cell and adsorbed onto the zeolite. The excess of pyridine was removed in vacuum by heating for 1 h at 423, 523, and 623 K, respectively. After each heating period, the temperature was reduced to room temperature and an IR spectrum was recorded. All the spectra were scaled according to the sample weight. The amount of carbon deposited in the catalysts after SRE was determined by elemental analysis using a Carlo Erba 1106 analyzer. ^27^Al MAS NMR spectra were recorded at 104.2 MHz with a spinning rate of 10 kHz and 9° pulse length of 0.5 µs with a 1 s repetition time in a Bruker AV 400 MAS spectrometer. ^27^Al chemical shifts were referred to Al^3+^(H_2_O)_6_.

### 2.3. Catalytic Study

SRE conditions were H_2_O/EtOH molar ratio of 13, atmospheric pressure, the value of weight hourly space velocity (WHSV) in this study was 0.95 h^−1^ (WHSV is defined as the weight of feed flowing per unit weight of the catalyst per hour), and a range of temperatures between 673 K and 873 K. The catalysts were reduced with hydrogen (100 mL/min) at 873 K for 2 h before reaction. The reactor was loaded with 0.3 mL of catalyst, weight 0.2 g, (grain-size: 0.25–0.42 mm), diluted with 3 g of carborundum (SiC) (grain-size: 0.60–0.80 mm). The water/ethanol mixture was fed from a pressurized container using a liquid flow controller (Bronkhorst) and vaporized at 473 K into a stream of nitrogen.

The analysis of the products of reaction was carried out online using a gas chromatograph (Varian 3800) equipped with two columns (TRB-5, L = 30 m, DI = 0.25 mm; CarboSieve SII, L = 3 m, DI = 2.1 mm) and two detectors, thermal conductivity (TCD), and flame ionization (FID). Equations (1) and (2) show the ethanol conversion and selectivity to the different reaction products, where (*F_EtOH_*)_0_ is the flow of ethanol fed to the reactor (mol·s^−1^), (*F_EtOH_*)*_f_* the flow of ethanol that comes from the reactor, and *F_j_* the flow of product j that comes from the reactor. Selectivity values were calculated excluding water.

(1) $$X_{EtOH}\;\left(\%,\;mol\right)=\;\frac{{\left(F_{EtOH}\right)}_{0}-{\left(F_{EtOH}\right)}_{f}}{{\left(F_{EtOH}\right)}_{0}}\times 100$$

(2) $$S_{j}\;\left(\%,\;mol\right)=\;\frac{F_{j}}{{\left(\sum^{}F_{j}\right)}_{products}}\times 100$$

## 3. Results and Discussion

### 3.1. Characterization

**Figure 1:**
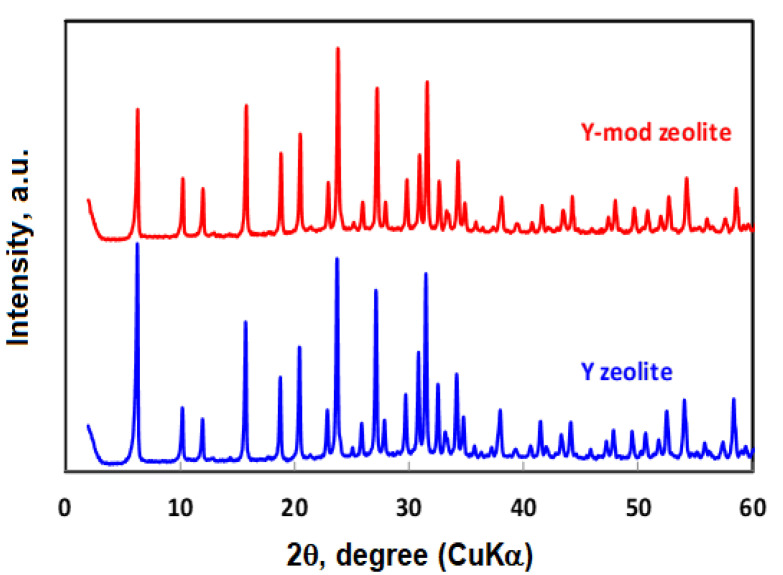
XRD of calcined Y and Y-mod zeolites.

The XRD of the calcined Y and Y-mod zeolites without cobalt are shown in Figure 1. As it can be seen, both zeolites show a similar diffraction pattern, indicating that the treatment to generate mesoporosity does not have a significant effect on the zeolite structure. Nevertheless, a slight decrease in the intensity of the diffraction peaks for the Y-mod zeolite is observed, which would be related to a small loss of crystallinity due mainly to the fact that the dealumination and desilication processes could not be occurring with the same intensity. Indeed, when the Si/Al ratio for these zeolites is determined, values of 2.5 for the pattern zeolite and 3.9 for the Y-mod (Table 1) were found, indicating that treatment seems to be slightly more selective for Al removal. This effect has been already described by Quin et al. for MFI-Type zeolite, where aluminum extra-framework was initially removed and after-framework Al and Si were extracted indiscriminately [41].

Table 1 shows the textural properties of the zeolitic materials and their corresponding cobalt catalysts. As it can be seen, the mesopore volume of the Y-mod zeolite is larger than that observed for the pattern Y zeolite. This fact seems to indicate that mesoporosity has been generated after dealumination/desilication treatment.

**Figure 2:**
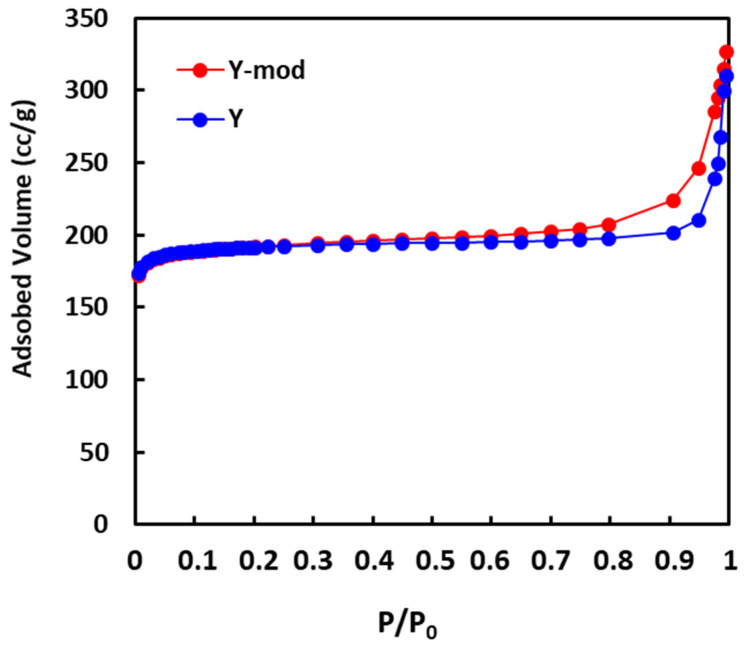
Adsorption isotherms of N_2_ of Y zeolite and Y-mod zeolite.

Mesoporosity can also be detected by the adsorption isotherms of N_2_. As it can be seen in Figure 2 in both samples, parent Y and Y-mod zeolites, the isotherms show nearly horizontal adsorption and desorption branches coupled with small hysteresis loops in the 0.5−1.0 partial pressure (P/P0) range. For Y-mod zeolite, a higher adsorption of N_2_ is observed compared with Y zeolite, suggesting the presence of pores with diameters in the range of mesopore for the sample subjected to dealumination/desilication treatment.

**Figure 3:**
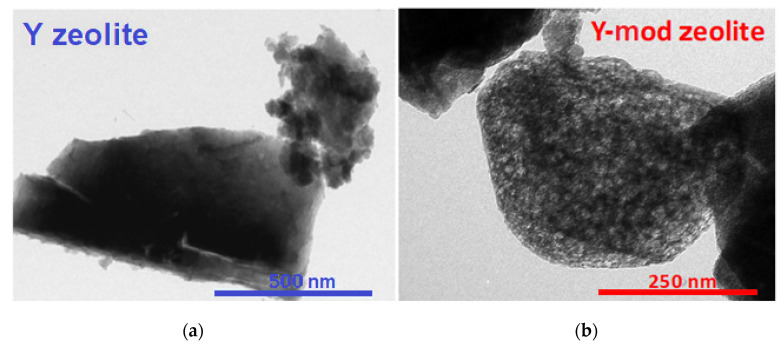
TEM microphotographs of Y zeolite (**a**) and Y-mod zeolite (**b**).

The formation of mesoporosity has been definitively confirmed by TEM. Figure 3 shows the TEM images of the Y and Y-mod zeolites. As it can be seen, Y-mod zeolite presents small brightnesses, which would confirm the presence of mesopores in its structure.

**Figure 4:**
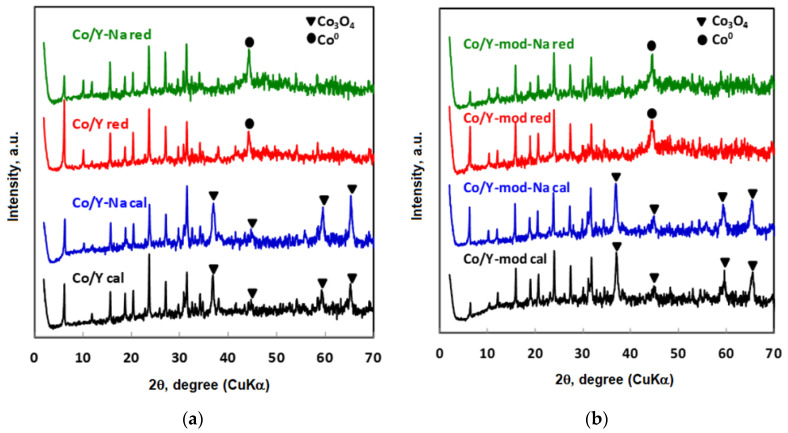
XRD of calcined and reduced Co catalysts (reduction conditions: 100 mL/min H_2_ at 873 K for 3 h). (**a**) Catalysts prepared using the pattern Y zeolite as support. (**b**) Catalysts prepared using mesoporized Y zeolite (Y-mod) as support.

Figure 4 shows the X-ray diffractograms of the calcined and reduced catalysts. Specifically, Figure 4a corresponds to the cobalt catalyst supported on the pattern Y zeolite (Co/Y) and Figure 4b corresponds to the cobalt catalyst supported on mesoporized Y zeolite (Co/Y-mod). As it can be seen for the calcined and reduced catalysts, the most part of the diffraction peaks is characteristic of the Y zeolite structure. For the calcined samples (bottom of Figure 4), additional diffraction peaks corresponding to Co_3_O_4_ (JCPDS 00-001-1152) [42,43,44,45] can be found. For the reduced catalysts (top of Figure 4), the main diffraction peaks related to the cobalt oxide (Co_3_O_4_) disappear, and appears the diffraction peaks corresponding to metallic cobalt (JCPDS 00-015-0806) as a consequence of the reduction process carried out [46,47,48,49,50].

Table 1 also shows the textural properties of the cobalt-based catalysts studied in this work. Therefore, the effect of the Co and Na, incorporated by incipient wetness impregnation and ionic exchange, respectively, on the textural properties of the final catalysts is presented in this Table. The obtained results show the presence of Co and Na decreases the BET surface area and the micropore and mesopore volume of the final catalysts. This result would be related to the partial blockage of the pores and mesopores of the zeolitic supports when Co and Na are incorporated. In addition, the high amount of incorporated cobalt (20 wt. %) could be also responsible of a dilution effect, which could be also contributing to the observed decrease of the pore volume.

The size of the cobalt metallic particles has been determined by the XRD of the reduced catalysts using the Scherrer equation [51]. As it can be seen in Table 2, the size of the Co^0^ clusters is smaller and the dispersion is significantly higher for the catalysts prepared with the zeolite Y-mod as support. This circumstance does not seem to occur for the cobalt supported on the pattern Y zeolite, without mesoporosity, where the sizes of the metallic particles are clearly larger. This result seems to show a positive effect of mesoporosity on the size and dispersion of metallic particles of cobalt.

**Figure 5:**
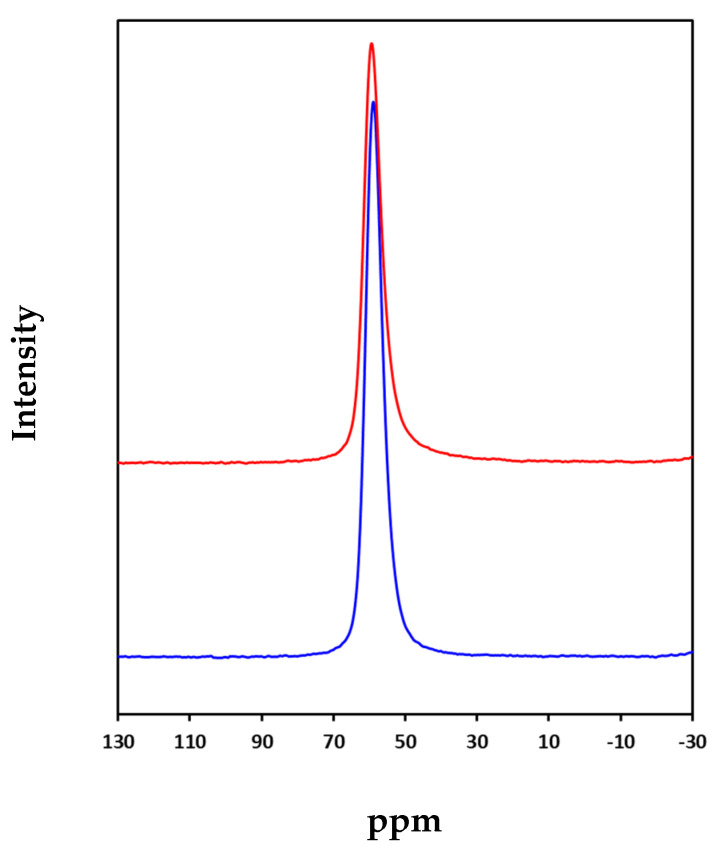
^27^Al MAS NMR spectra of Y and Y-mod zeolites.

The acidity of the samples has been studied by adsorption-desorption of pyridine [52]. As it can be seen in Table 3, the parent Y zeolite and its mesoporized derivative prepared by NH_4_F contain mainly Brønsted acid sites. This result is consistent with the ^27^Al MAS NMR spectra presented in Figure 5, where a single peak at 55 ppm, corresponding to tetrahedral aluminum, is observed in both samples, parent and mesoporized Y zeolite.

For the samples containing Co (Co/Y and Co/Y-mod), the Lewis acidity increases significantly, while Brønsted acidity decreases considerably. This decrease in Brønsted acidity and rise in Lewis has already been described in zeolites when transition metals as cobalt are incorporated [53]. Finally, the acidity of the catalysts exchanged with Na was neutralized since Brønsted acidity was not detected. Therefore, the Brønsted acidity decreases as follows in the catalysts: Co/Y-mod > Co/Y >>> Co/Y-Na = Co/Y-mod-Na.

**Figure 6:**
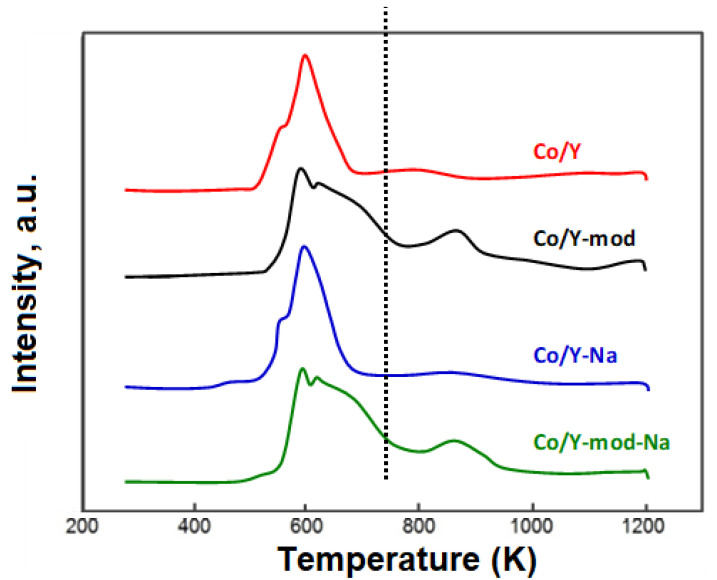
Temperature-programmed reduction (TPR) of calcined catalysts. (---) Reduction temperature of the catalysts before reaction (873 K).

The reducibility of the Co-based catalysts has been studied by TPR. Figure 6 shows that the four catalysts present two reduction peaks around 550 K and 590 K, which correspond to the reduction of the oxidized Co species to metallic cobalt in two stages. The first one corresponds to the transition from Co_3_O_4_ to CoO and the second to the transition from CoO to Co^0^ [42,54,55,56]. The catalysts based on Y-mod show a third peak at higher reduction temperatures (680–1050 K), indicating the existence of cobalt species interacting strongly with the support. These cobalt species could be assigned to the reduction of cobalt silicates probably formed during the calcination stage [42,57,58]. These results seem to indicate that cobalt oxides exhibit higher interaction with modified Y zeolite and could be related to the different size of the Co metallic particles found in each support (Table 2). Smaller particle size would be related to stronger metal-support interactions, explaining the reduction peaks found at higher reduction temperatures. Indeed, the highest amount of hydrogen adsorbed at high temperatures were detected for Co/Y-mod and Co/Y-mod-Na catalysts, which exactly showed the smallest Co metallic particle sizes. Considering that the catalysts before reaction are reduced at 873 K, it is possible that a part of the cobalt species in the catalysts remains in its oxidized form. The degree of reduction of the oxidized Co species present in the catalysts at 873 K has been determined from the TPR profiles (Table 2). In general, the reducibility of the oxidized species of Co in the catalysts at 873 K is remarkably high. The lowest reducibility value is 88%, corresponding to the Co/Y-mod catalyst, and the highest value is 95%, corresponding to the Co/Y catalyst. In the case of the catalysts prepared with Y zeolite subjected to the dealumination/desilication process, it can be seen that they present a slightly lower degree of reduction compared to the catalyst prepared with untreated Y zeolite.

### 3.2. Catalytic Activity

Table 4 shows the ethanol conversion and selectivity to the different products obtained in the steam reforming for the four catalysts studied in this work. As it can be seen, ethanol conversion is extremely high for all the catalysts, almost complete, at least at the temperature range here studied (773–873 K). However, in terms of selectivity, important differences can be found. As it can be seen, Co/Y and Co/Y-mod catalysts present a high selectivity to ethylene and a low production of hydrogen. This effect is more prominent in the catalyst based on Y-mod. These results could be related to the acidity presented by these samples (Table 3). It is well known that the presence of acid sites favors the reaction of dehydration of ethanol [29,39], explaining the high concentration of ethylene produced by the Co/Y and Co/Y-mod samples. Specifically, the largest production of ethylene observed in the sample based on Y-mod could be due to the higher accessibility of its acid sites, probably favored by the presence of mesopores. On the contrary, the highest production of hydrogen by the neutralized catalysts (Co/Y-Na and Co/Y-mod-Na) suggests that the presence of acid sites is not advantageous to produce hydrogen via steam reforming of ethanol [29,39]. In addition, the larger size of the cobalt metal particles present in the Y zeolite with Na (Co/Y-Na), would explain its higher production of acetaldehyde, methane, and CO compared to the Co/Y-mod-Na catalyst, whose sizes are much smaller [31]. Large metallic particles would lead to a decrease of the active metal surface and thus, to a decrease in the number of the active sites where the reforming reaction can occur. This fact could explain the high selectivity to H_2_ presented by the Co/Y-mod-Na sample, which contains smaller metallic cobalt particles. These results seem to indicate that the presence of mesopores could help to promote a higher interaction of cobalt with the support, resulting in the formation of smaller and more dispersed metallic Co particles. Therefore, if small metal particles were presented, a larger number of active sites of Co would be available for the steam reforming of ethanol and for all those secondary reactions related to the selective production of H_2_.

Finally, carbon deposition on the exhausted catalysts were determined to know their stability. It is well known that the formation of ethylene is directly related to the coke formation and the presence of coke directly related to the stability of one catalyst [14,59,60]. At first glance, the catalysts after reaction showed a characteristic black color, which suggests the presence of coke. Elemental analysis technique was used to determine quantitatively the amount of deposited coke. Table 5 shows that the samples containing Na (Co/Y-Na and Co/Y-mod-Na) present a lower amount of coke than the catalysts without Na (≈20 wt. % against ≈30 wt. %). The larger coke deposition observed in the catalysts not subjected to ionic exchange with Na (Co/Y and Co/Y-mod) could be explained by the greater acidity of theses samples and the consequences that this entails: increasing of the dehydration reaction and generation of high amounts of ethylene, an important coke precursor.

Table 5 shows also that the Co/Y-Na catalyst presents a higher carbon content compared to the Co/Y-mod-Na catalyst. In both catalysts, the acidity was neutralized. Therefore, it is foreseeable that the mesoporosity present in the Co/Y-mod-Na catalyst could provide a positive effect to decrease the formation of carbon. This fact could be explained in part considering the better diffusion of reagents and products during the SRE [29]. In addition, mesoporosity seems to promote the formation of cobalt metallic particles of smaller size, which seem to also decrease the formation of coke and coke precursors during the steam reforming of ethanol [27,36]. In summary, the results here showed seem to indicate that it is possible to improve the activity and stability of a cobalt bioethanol steam reforming catalyst supported on Y zeolite through the generation of mesoporosity and the neutralization of its acid sites.

## 4. Conclusions

The effect of mesoporosity and acidity of a Y zeolite-based catalyst promoted with Co has been studied in the steam reforming of ethanol. The results obtained with the “mesoporized” Y zeolite and neutralized with Na show a high activity, selectivity, and low coke deposition. The results of the physicochemical characterization suggest that the mesoporosity generated and the neutralization of the acid sites by Na exchanged would be primarily responsible of the good performance exhibited by the cobalt catalyst supported on Y zeolite, due to the lower acidity and the smaller size of the metallic Co particles present in this sample.

## Figures and Tables

**Table 1:** Textural properties of the catalysts and supports studied in this work.

Catalyst	Si/Al	Na (wt. %)	BET Area (m^2^/g)	Mesopore Volume (cm^3^/g)	Micropore Volume (cm^3^/g)
Y	2.4	0.21	629	0.034	0.292
Y-mod	3.9	0.11	544	0.110	0.244
Co/Y	2.3	0.15	499	0.036	0.241
Co/Y-mod	3.8	0.06	409	0.080	0.180
Co/Y-Na	2.4	3.13	406	0.020	0.190
Co/Y-mod-Na	3.9	4.21	371	0.037	0.146

**Table 2:** Co content, crystal size of metallic Co (XRD), dispersion of metallic Co, and percentage of Co reduced at 873 K (reducibility).

Catalyst	Co (wt. %)	Co^0^, XRD (nm)	Co^0^ Dispersion (%) ^a^	Reducibility % (873 K)
Co/Y	19.1	29	3.3	95.4
Co/Y-mod	19.0	14	6.9	88.3
Co/Y-Na	19.3	28	3.4	94.2
Co/Y-mod-Na	18.7	13	7.4	89.1

^a^ Calculated from dCo(nm) = 96/D(%), D: dispersion and dCo(nm): size of the Co metallic particle determined by XRD.

**Table 3:** Acidity of the catalysts and supports studied in this work.

Catalyst	B (mmol py)			L (mmol py)		
	523 (K)	623 (K)	673 (K)	523 (K)	623 (K)	673 (K)
Y	0.254	0.127	0.076	0.034	0.023	0.023
Y-mod	0.235	0.127	0.029	0.070	0.061	0.018
Co/Y	0.010	0.005	0.004	0.777	0.312	0.140
Co/Y-mod	0.103	0.025	0.019	0.861	0.385	0.249
Co/Y-Na	-	-	-	0.532	0.276	0.127
Co/Y-mod-Na	-	-	-	0.671	0.302	0.177

**Table 4:** Ethanol conversion and products selectivity in steam reforming of ethanol for the catalysts studied in this work. (Reaction conditions: H_2_O/EtOH = 13, WHSV 0.95 h^−1^ and atmospheric pressure).

Catalyst	T (K)	Ethanol Conv. % mol	Selectivity % mol					
			CH_4_	CO	CO_2_	H_2_	C_2_H_4_	C_2_H_4_O
Co-Y	673	98.5	1.1	4.3	4.7	17.5	71.6	0.7
	773	99.8	1.7	3.4	21.4	39.0	32.7	1.7
	873	99.9	3.4	11.2	22.2	43.5	18.7	1.0
Co-Y mod	673	99.9	0.0	0.0	0.3	0.2	95.8	3.6
	773	100	0.6	1.0	0.9	13.9	78.7	4.6
	873	99.8	2.2	5.0	2.3	25.7	59.6	4.9
Co-Y Na	673	94.7	7.0	7.0	14.2	68.9	0.6	2.2
	773	95.9	3.7	5.1	18.1	70.3	0.8	1.0
	873	99.9	4.6	11.6	12.0	69.7	0.8	1.2
Co-Y mod Na	673	99.8	4.9	6.0	15.2	73.3	0.2	0.4
	773	99.9	3.0	2.6	20.3	74.0	0.1	0.0
	873	99.9	2.0	4.3	19.5	74.1	0.0	0.0

**Table 5:** Carbon deposition of the catalysts after 34 h of reaction time. (Reaction conditions: H_2_O/EtOH = 13, WHSV 0.95 h^−1^ and atmospheric pressure).

Catalyst	Carbon (wt. %)
Co/Y	31.3
Co/Y-mod	34.7
Co/Y-Na	24.8
Co/Y-mod-Na	19.1
